# Patient experiences of PMR: a qualitative narrative literature review

**DOI:** 10.1093/rap/rkag006

**Published:** 2026-01-16

**Authors:** Max Yates, Janice Mooney, Pratyasha Saha, Claire E Owen, Sarah L Mackie, Louise Falzon, Aatke Van der Maas, Thomas Bolhuis, Philipp Bosch, Christian Dejaco, Lorna Neill, Marie McGee

**Affiliations:** Norwich Medical School, University of East Anglia, Norwich, UK; Department of Rheumatology, Norfolk and Norwich University Hospital, Norwich, UK; School of Health and Social Care, University of Staffordshire, Stafford, UK; Norwich Medical School, University of East Anglia, Norwich, UK; Department of Rheumatology, Norfolk and Norwich University Hospital, Norwich, UK; Department of Medicine, The University of Melbourne, Parkville, Victoria, Australia; Department of Rheumatology, Austin Health, Heidelberg, Victoria, Australia; Leeds Institute for Rheumatic and Musculoskeletal Medicine, University of Leeds, Leeds, UK; Leeds Biomedical Research Centre, Leeds Teaching Hospitals NHS Trust, Leeds, UK; School of Medicine and Population Health, University of Sheffield, Sheffield, UK; Rheumatology, Sint Maartenskliniek, Ubbergen, The Netherlands; Rheumatology, Sint Maartenskliniek, Ubbergen, The Netherlands; Department of Rheumatology, Medical University of Graz, Graz, Austria; Department of Rheumatology, Medical University of Graz, Graz, Austria; Department of Rheumatology, Hospital of Bruneck (ASAA-SABES), Brunico, Italy; Department of Rheumatology, Teaching Hospital of the Paracelsius Medical University, Brunico, Italy; Patient Charity Polymyalgia Rheumatica and Giant Cell Arteritis Scotland, Dundee, UK; School of Health Sciences, University of East Anglia, Norwich, UK

**Keywords:** PMR, patient experiences, diagnosis, glucocorticoids

## Abstract

**Objectives:**

PMR is a common inflammatory condition characterized by pain and stiffness in the shoulders and hips. Patient experiences of PMR remain underexplored and often diverge significantly from clinician perspectives, contributing to the overall burden of the disease. This review forms part of an ongoing project conducted by the PMR Working Group of Outcome Measures in Rheumatology (OMERACT) with the aim of exploring patient views of ‘relapse’ and ‘remission’.

**Methods:**

A comprehensive search was conducted across four electronic databases (Ovid MEDLINE, Ovid EMBASE, The Cochrane Library and CINAHL) from database inception to 31/01/2025, to identify qualitative studies reporting patient experience in PMR. Study quality was appraised using the Critical Appraisal Skills Programme qualitative tool, and thematic synthesis used to integrate findings.

**Results:**

Five studies met inclusion criteria and thematic analysis revealed three overarching themes: (1) the pathway to diagnosis, (2) managing uncertainty and (3) challenges to everyday life. Subthemes provided deeper insights into patient experiences, including delays in help-seeking due to the rationalization of symptoms, and complex responses to glucocorticoid treatment, described by participants as a ‘double-edged sword’, offering rapid improvement in symptoms but also causing significant distress. Notably, commonly used clinical terms such as ‘relapse’ and ‘remission’ were often inconsistent with how patients described their own experiences, underscoring a gap between clinical definitions and patient experiences.

**Conclusion:**

This qualitative narrative literature review reveals the unique challenges of disease management and the complex realities of long-term glucocorticoid use. These findings highlight the urgent need for more patient-centred approaches to care and support.

Key messages

- Patients often delay seeking care by normalizing symptoms.
- Glucocorticoids rapidly relieve PMR symptoms but cause distressing side effects and emotional uncertainty for patients.
- Clinical terms like relapse and remission often misalign with patient experiences, requiring more patient-centred language.

## Introduction

PMR is a common inflammatory disease that presents with muscle pain and stiffness, usually at the shoulders and the hips, and is associated with elevated systemic inflammatory markers. Its prevalence in the UK is 1.7% in individuals over 55 years with increasing incidence with age [1, 2]. Furthermore, PMR disproportionately affects women who comprise two-thirds of cases [1, 2].

Although PMR is common, treatment advances have been limited, and new biological therapies are presently only licensed in the USA for those with relapsing disease [3–8]. Glucocorticoids (‘steroids’) remain the therapeutic mainstay, most commonly prednisolone. Although glucocorticoids can rapidly improve initial disease control, patients commonly experience worsening symptoms upon tapering the dose or following complete cessation, which is commonly referred to as ‘relapse’ or ‘flare’, with periods of disease control in between referred to as ‘remission’. Escalation of glucocorticoid dose and high cumulative prednisolone exposure invariably ensues, leading to well characterized adverse events such as infection, hypertension, diabetes and osteoporosis [9–13]. Furthermore, adverse effects of importance to patients are frequently reported, including weight gain, face and neck changes, sleep disturbance and skin fragility, yet their impact on quality of life is seldom measured in research studies [14].

Survey data from patients and general practitioners (GPs) have identified that there is an ad-hoc approach to steroid prescribing in PMR, due to challenges in diagnosis and management, with patients often left to self-manage their steroid taper [15]. Conflict can arise due to differing views between patients and their treating physicians as to what constitutes a ‘relapse’, and many patients struggle with steroid reduction due to the increased risk and fear of recurring symptoms [16]. Although most patients with PMR express a strong desire to reduce and stop steroids, pain, stiffness and limitation of mobility are the primary driver for continued steroid usage [17].

Notably, around 70% of PMR patients in the UK are managed exclusively within primary care, with specialist input sought only for complex or atypical cases from rheumatologists, geriatricians, nurse specialists and physiotherapists [1, 18]. However, this poses challenges to patient management due to the uncertainties associated with the disease which include the unpredictability of the disease course and the management of ‘flares’, combined with long-term steroid adverse effects [19, 20]. Moreover, patients’ experiences of PMR, their understanding of ‘relapse’ and ‘remission’, and their use of steroid therapy is captured poorly and so understanding of these terms may differ [14].

Prior literature has suggested a mismatch between patients and physicians in the terminology used for the diagnosis and management of PMR. For example, patients describe ‘weakness’ as a symptom, but this was not recognized by physicians, while physicians used ‘morning stiffness’ as a measure of disease activity, although patients often reported stiffness lasting throughout the day [20]. Patients also report difficulty in managing uncertainty and managing other peoples’ expectations and recommendations relating to steroid therapy [19]. Understanding patient experience could therefore facilitate a shared understanding between the clinician and the patient when faced with the challenges of glucocorticoid dose adjustment.

### Aim

The aim of this study was to carry out a narrative literature review of studies that explored patient experiences of PMR to inform how these referred to ‘relapse’ and ‘remission’, terms commonly used by clinicians but without an agreed definition. This work is part of a broader Group of Outcome Measures in Rheumatology (OMERACT) project by its PMR Working Group to develop ‘relapse’ and ‘remission’ criteria. Insights from the analysis of literature concerning the patient experience in PMR are anticipated to help clinicians better understand individual needs and to inform the development of outcome measures that support a more personalized and holistic approach to PMR assessment and management.

## Methods

### Literature search strategy

A comprehensive search strategy was conducted to explore the lived experiences of ‘relapse’ and ‘remission’ in PMR. A professional librarian carried out a search of the qualitative research literature across Ovid (Medline), EMBASE, The Cochrane Library and CINAHL from database inception to 31/01/2025.

The following search terms were used: (‘polymyalgia rheumatica’ or ‘giant cell arteritis’) AND (relapse or remission or rehospitali*) AND (experience or qualitative or phenomenolog* or survey or interview* or questionnaire*).

All titles and abstracts were screened by three independent reviewers (M.Y., J.M., M.M.). Full-text papers were identified in accordance with eligibility criteria. Any discordance regarding study eligibility was resolved by discussion. Reference lists of the included studies were additionally hand searched to identify additional studies but none were identified.

### Inclusion and exclusion criteria

**Figure 1:**
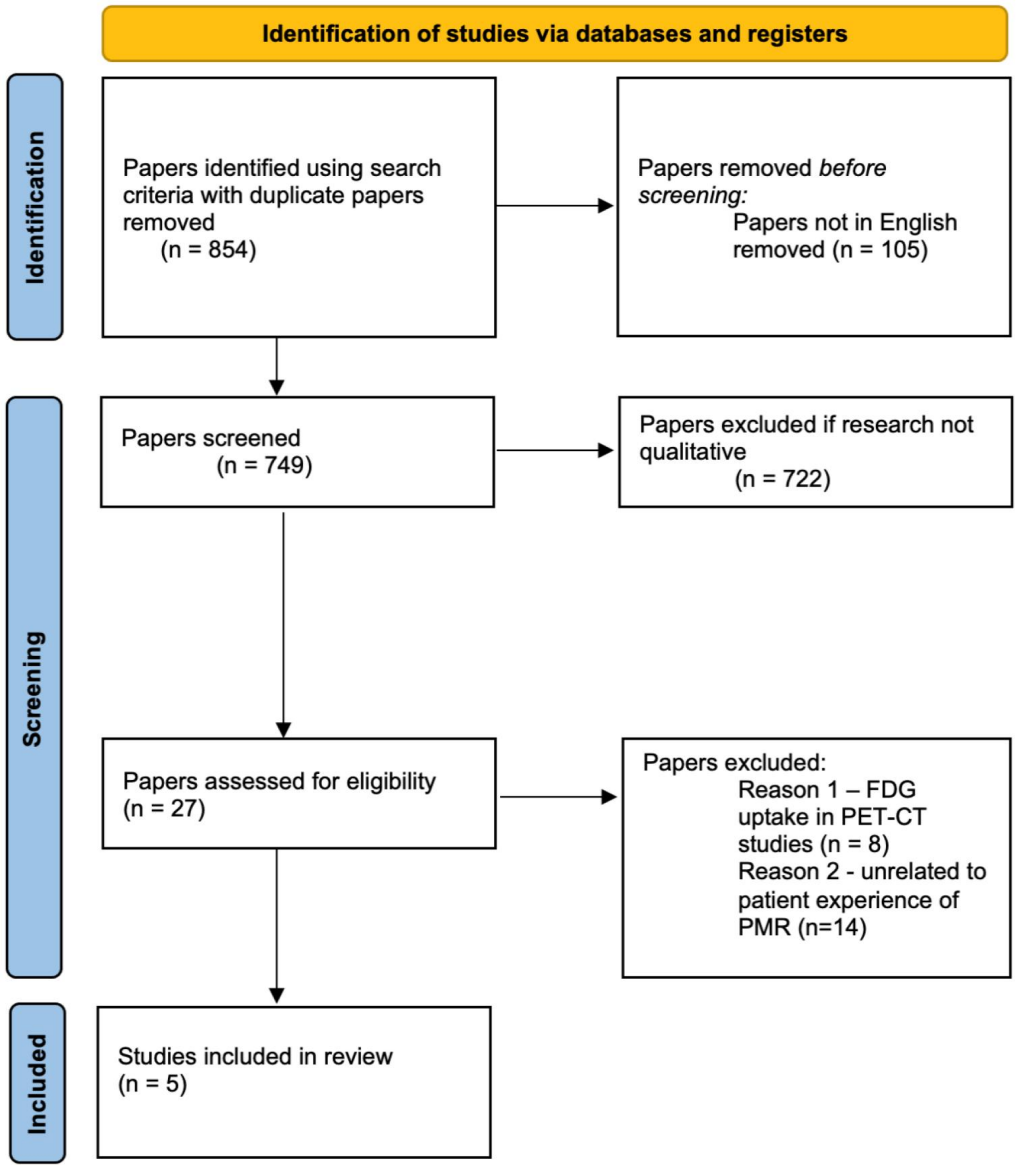
PRISMA 2020 flow diagram. PRISMA flow diagram adapted from Page et al. [21]. FDG: fluorodeoxyglucose; PET-CT: positron emission tomography—computed tomography

Original research articles published in English with full text versions were included. Inclusion criteria were diagnosis of PMR and an explicit focus on patient experience of the condition. While ‘giant cell arteritis’ (GCA) was included in the search terms, the intention was to include them if the study subsequently explored specific experience of PMR, but none were identified. Exclusion criteria were papers that did not focus on the patient experiences of PMR. The Preferred Reporting Items for Systematic reviews and Meta-Analyses (PRISMA) flowchart, detailing the steps involved in the article selection process [21], is shown in Fig. 1.

### Quality appraisal

The Critical Appraisal Skills Programme (CASP) 2025 qualitative checklist was used to appraise the quality of the four studies with all scores indicating high quality (see Supplementary Table S1, available at *Rheumatology* Online). One study did not fully meet the criteria for formal CASP appraisal due to its non-traditional format and limited reporting. However, as the authors did provide some methodological detail regarding participant recruitment, data collection and ethical approval, given the limited availability of qualitative research in PMR, this source was deemed relevant to the review question.

### Data extraction and synthesis

A standardized data extraction template was developed to ensure consistency across studies. Extracted information included study characteristics (author, year, country, setting, sample characteristics, data collection method and analytical approach) and key findings relevant to patient experiences of PMR (Table 1).

**Table 1:** Overview of included studies.

**Author (year)** **Title, journal, country**	Methods	**Study setting** **Case definition**	Themes	Limitations
Twohig et al. (2015) [20] “I suddenly felt I’d aged”: A qualitative study of patient experiences of polymyalgia rheumatica (PMR). Patient Educ Couns UK	Qualitative semi-structured interviews of 22 patients with PMR (10 females, 12 males) following topic guide. Constant comparative method used to identify themes with use of Nvivo10 software.	Cases recruited from 10 South Yorkshire general practices selected through purposive sampling for diverse practices Cases >50 years old with a read coded PMR diagnosis and classical PMR symptoms documented in electronic medical record	Five themes identified Pain, stiffness and weakness Disability Treatment and disease course Experience of care Psychological impact of PMR	More men than women recruited; primary care setting—no participants from secondary care; GP carried out the interviews
Mackie et al. (2015) [17] ‘An impediment to living life’: why and how should we measure stiffness in polymyalgia rheumatica? PLoS One UK	Qualitative interviews of 50 patients with PMR through 8 focus groups (36 females, 14 males). Each focus group with one facilitator and one rapporteur following common interview schedule. Six stages of inductive thematic analysis performed, with focus on ‘stiffness’ symptom.	Cases recruited from three geographically separated centres in England selected through purposive sampling aiming for diversity of age, gender and disease duration Cases with current or previous PMR diagnosed by a rheumatologist	Four themes identified Symptoms: pain, stiffness and fatigue Functional impact Impact on daily schedule Approaches to measurement	Recruitment solely from secondary care centres, not from primary care
Tshimologo et al. (2017) [24] Patients’ views on the causes of their polymyalgia rheumatica: a content analysis of data from the PMR cohort study BMJ Open UK	Self-completion questionnaire of 654 PMR patients (408 females, 246 males). Summative content analysis approach of written answer to question ‘What do you think caused your PMR?’	Cases recruited from primary care PMR inception cohort, from 382 GP practices across England Cases with recent PMR, diagnosed by GPs who were provided the British Society for Rheumatology (BSR) guidelines	Three themes of patient beliefs for proposed causes of PMR Ageing process Personal stress Medication	This is one question analysed from a larger questionnaire; does not allow for in-depth exploration of patients’ beliefs of what cause their PMR
Hoon et al. (2019) [19] A qualitative study of patient perspectives related to glucocorticoid therapy in polymyalgia rheumatica and giant cell arteritis Open Access Rheumatol Australia	Qualitative semi-structured interviews of 14 patients with PMR through 4 focus groups or telephone interviews (9 females, 5 males) following a script, with a pre-focus group questionnaire. Framework method used for phenomenological analysis with use of Nvivo10 software.	Cases from two tertiary Australian hospital rheumatology clinics Cases were current patients with PMR and/or GCA diagnosed by a rheumatologist—all selected cases in the paper had PMR without GCA	Four themes identified Side effects of steroids Impact of steroids side effects on quality of life Managing uncertainties of condition Managing recommendations related to steroid therapy	Recruitment solely from tertiary centres, not from primary care
Harris et al. (2023) [22] Exploring the patient experience in polymyalgia rheumatica Clin Rheumatol Australia	Qualitative semi-structured interviews of 15 patients with PMR through 5 online focus groups, with a pre-focus group survey completed by 95 patients. As this work was published as a letter to the editor, subsequent full anonymized transcripts were analysed using thematic analysis.	Cases from two Australian hospital rheumatology clinics Cases were patients with PMR diagnosed by a rheumatologist	Five themes identified Symptoms affecting quality of life Delay to diagnosis Attitude to steroid treatment Side effects of steroid treatment Desire for alternate treatment options	The methods for data analysis and selection were not clear. This was not a traditional qualitative source, which had an impact on reporting of design and study findings

GP: general practitioner.

Data were extracted from four qualitative studies and one letter to the editor containing relevant patient narratives. Owing to the limited research exploring patient lived experiences of PMR, additional anonymized transcript data were obtained from the authors of the letter (Harris et al. [22]) with ethical permissions and institutional approvals to enhance the synthesis.

Both first-order constructs (participants’ direct quotes and narrative accounts) and second-order constructs (authors’ interpretations and reported themes) were extracted and treated as qualitative data for synthesis. Thematic analysis followed Braun and Clarke’s six-phase framework for reflexive thematic analysis [23]. The process involved familiarization with the data through repeated reading, inductive generation of initial codes representing meaningful features and iterative refinement through constant comparison across studies. Codes were then collated into potential themes, which were reviewed, defined and named to capture shared patterns of meaning and experience while preserving contextual and interpretive nuance.

### Ethics

This study is a qualitative narrative review and did not involve the collection of new data. In accordance with our institution’s ethical guidelines, approval was obtained (ETH2526-0324) for the secondary analysis of the published data from the study by Harris et al. [22]. Ethical permission to access and use the anonymized data for secondary analysis was granted by the original study authors with consent obtained from participants before the data were reused. Anonymized data sharing was carefully managed in collaboration with the original research team and institutional ethics guidance.

## Results

### Included studies

Five studies were included in the final thematic analysis (Fig. 1) following literature search and appraisal; three studies were based in the UK and the other two in Australia.

### Included study methods

Table 1 summarizes the five papers that underwent thematic synthesis in this qualitative narrative review. As an overview, the study by Twohig et al. [20] used semi-structured interviews to discover patient experiences of PMR; Mackie et al. [17] used focus groups to explore patients’ concepts of stiffness in PMR, and how they think stiffness should be measured; Tshimologo et al. [24] explored patient’s beliefs about the causes of their PMR using written responses to a questionnaire; Hoon et al. [19] used focus groups and telephone interviews to explore individuals’ experiences of living with PMR; and Harris et al. [22] used semi-structured interviews and a survey to find out patients’ perspectives of PMR.

### Thematic analysis

**Figure 2:**
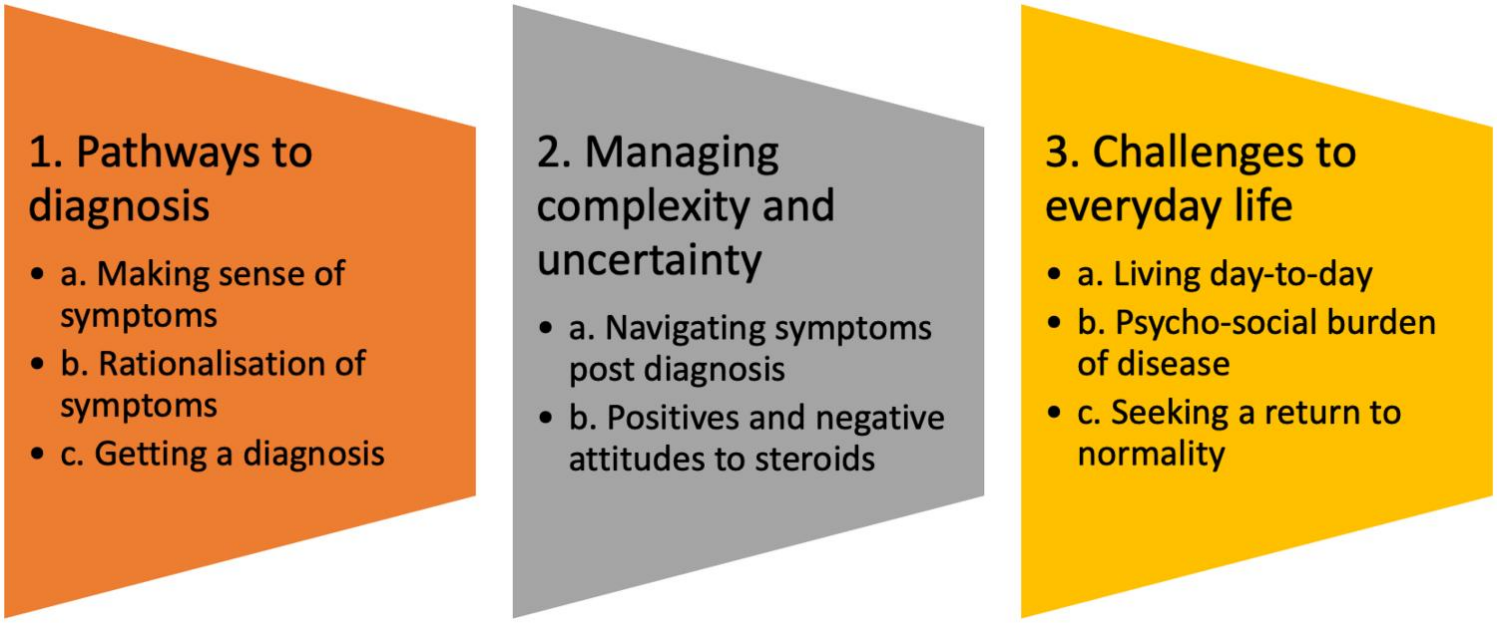
Themes and subthemes for the patient experience of PMR

Three main over-arching themes were identified within the published work through thematic synthesis, with additional subthemes explored below (Fig. 2).

1. Pathway to diagnosis
2. Managing uncertainty
3. Challenges to everyday life.

### Theme 1: pathways to diagnosis—‘I think something’s really wrong’

Participants described varied and often difficult journeys to diagnosis, shaped by how they interpreted and responded to their symptoms. While a few received prompt and accurate diagnoses, many experienced significant delays as symptoms were mistaken for other conditions or attributed to ageing (Mackie et al. [17]; Twohig et al. [20]; Harris et al. [22]). The process of making sense of unexplained pain and stiffness was marked by uncertainty, self-doubt and fear.

#### Making sense of symptoms—‘I’ve never had anything like it ever’

The abrupt onset of symptoms left many participants alarmed and confused, recognizing that something was seriously wrong. As one participant described, *‘If you can’t get out of bed to go to the toilet, there’s something seriously wrong with you, isn’t there?’* (Mackie et al. [17]). Others described the sudden loss of physical function: *‘I just woke up with it-I thought I’d had a stroke because I couldn’t lift my left arm’* (Harris et al. [22]).

Several participants initially downplayed their symptoms, attempting to continue daily routines until pain and fatigue became overwhelming. One woman described this turning point, *‘I became very unwell and very rundown. Still tried to maintain everything that I did because that’s just who I was. I noticed I couldn’t walk very well… and I woke up one morning. I said to my husband, “I can’t breathe. It felt like I had a brick on my chest. I think something’s really wrong. I need to go to the hospital”’* (Harris et al. [22]).

Some participants trusted their bodily awareness to signal that something was amiss: *‘When you get to my age and you’ve been fit, you know your own body, you know something’s right or wrong’* (Twohig et al. [20]). This intuitive recognition of change often legitimized their decision to seek medical help.

#### Rationalizing symptoms—‘Oh, it’s me age’

Despite significant discomfort, many participants normalized early symptoms, attributing them to ageing or minor illness. As one participant reflected, *‘I was aching more, really stiff, and I thought, “Oh, it’s just me age.” I’ll just sort of work through it. But then three months on… I was in bed, just agony’* (Twohig et al. [20]). Others made sense of their pain through familiar explanations such as osteoarthritis or infections, remarking: *‘My age, worn-out joints’,* and *‘I would suggest age is the major factor’* (Tshimologo et al. [24]).

Some associated onset with recent illness: *‘Pain started after a chest infection or flu jab’* (Harris et al. [22]). This tendency to rationalize or minimize symptoms often contributed to delayed help-seeking, with several describing a crisis point before presenting to emergency care: *‘Took myself to ED crying and begging them for help’* (Harris et al. [22]).

#### Getting a diagnosis—‘blood test after blood test, doctor after doctor’

For many participants, receiving a diagnosis was an exhausting and emotional process. Some experienced relief when their suffering was finally validated: *‘She printed sheets out and said, ‘This is exactly you,’ and it was-it was me, definitely me’* (Twohig et al. [20]). Others endured lengthy investigations and multiple consultations: *‘Blood test after blood test, doctor after doctor. Still no result. If I hadn’t taken it into my own hands, I still wouldn’t have a diagnosis’* (Harris et al. [22]).

Participants frequently felt dismissed or misunderstood: *‘And I went to the doctors, well they were telling me to take paracetamols like and then they were no good, he increased it to some stronger stuff and I went back again, I said ‘they weren’t doing us any good’* (Twohig et al. [20]). Family advocacy was sometimes needed to prompt further investigation: ‘*My husband went up “look my wife can’t get out of bed this morning”: I think you ought to send her to hospital. She needs treating, she’s not getting anywhere. And that’s what she did then, sent me to hospital’* (Twohig et al. [20]). The eventual diagnosis often brought relief but also frustration over the time lost: *‘The day they told me I’d got PMR I was euphoric’* (Harris et al. [22]).

Across studies, limited professional awareness of PMR and misattribution to common conditions delayed diagnosis and treatment, intensifying participants’ sense of anxiety and helplessness.

### Theme 2: managing complexity and uncertainty—‘Oh, am I going to be like this all the time?’

Following diagnosis, participants struggled with the unpredictable course of PMR and the complexities of managing glucocorticoid therapy. Relief at finally having an explanation was tempered by fear that symptoms would persist indefinitely and by uncertainty over how to balance symptom control with medication side effects (Mackie et al. [17]; Hoon et al. [19]; Harris et al. [22]).

#### Navigating symptoms post-diagnosis—‘good or bad day’

Many participants described daily life as a continual negotiation between ‘good’ and ‘bad’ days. As one participant expressed, *‘The pain was the most frightening thing I’d ever suffered. I thought, oh, am I going to be like this all the time?’* (Harris et al. [22]). Another noted, *‘A bad day for me would be when I have difficulty moving about, getting up from a chair, cooking’* (Mackie et al. [17]).

Participants learned to plan around symptom fluctuations: *‘My best time is ten o’clock till three, then I seem to get really tired… when you sit down, you kind of seize up’* (Twohig et al. [20]). Yet, the perceived lack of professional guidance left many feeling isolated. As one participant explained, *‘If I try and go down [the dose], I’ll get away with it for two or three weeks, then you think, is this because I’ve gone down or is this something new?’* (Hoon et al. [19]). The need to self-manage medication adjustments reinforced feelings of uncertainty and abandonment.

#### Attitudes to steroids—‘it was like magic’

Glucocorticoid treatment was often described as both life-changing and problematic—a ‘double-edged sword’. Participants clearly recalled the dramatic relief of initial therapy: *‘He put me on prednisolone and it was like magic… I can’t say to you what a difference this has made to me’* (Twohig et al. [20]). Others expressed gratitude but mixed emotions: *‘They do ease the pain, but they make me agitated and cause weight gain. I couldn’t do without them, but I don’t like them’* (Harris et al. [22]).

The impact of negative experiences was equally profound: *‘I had a terribly swollen face, which I’ve still got-a huge neck from the high doses of prednisolone’* (Hoon et al. [19]). One participant reflected, *‘Cortisone is a wonder drug with PMR, but the side effects of weight gain and irritability impact on you. A happy personality becomes snappy and anxious’* (Harris et al. [22]). The physical and psychological burden of treatment reinforced the sense of living with a condition that was simultaneously visible, misunderstood, and self-managed.

### Theme 3: challenges to everyday life—‘it’s a bit of a merry-go-round’

This theme captures the ongoing struggle to maintain normality while living with PMR. Persistent pain, stiffness and fatigue disrupted daily routines, restricted independence and shaped emotional well-being (Mackie et al. [17]; Twohig et al. [20]; Harris et al. [22]).

#### Living day-to-day—‘difficulty moving about’

Loss of mobility and function transformed participants’ lives. Ordinary tasks became major challenges: *‘A bad day for me would be when I have difficulty moving about, getting up from a chair, cooking’* (Mackie et al. [17]). Another participant recalled, *‘I didn’t know how to get in the car because my legs wouldn’t bend… I couldn’t lift my arms to comb my hair… really struggling with everything’* (Twohig et al. [20]). These physical limitations compromised independence, frequently leading to dependence on others for everyday care.

#### Psychosocial burden—‘you dread the morning to come’

Emotional distress accompanied the physical burden of PMR. Participants who had been previously active described feelings of helplessness and diminished self-worth: *‘I completely lost my drives, like my drives in a machine-complete lack of power’* (Harris et al. [22]). For some participants, feelings of vulnerability manifested in emotional breakdowns: *‘He started crying and said, “I’m bloody useless”’* (Twohig et al. [20]).

Despair and hopelessness were common, particularly during symptom flares: *‘You dread the morning to come. You don’t want to wake up’* (Mackie et al. [17]). These accounts highlight how PMR eroded participants’ sense of self, leaving them vulnerable to social isolation and anxiety.

#### Seeking a return to normality—‘I can live with it’

Despite ongoing limitations, many sought to adapt and regain control. For some, improvement through treatment enabled partial restoration of daily function: *‘I can put up with it, I can live with it… it’s not as much of a sharp pain now, just a nagging ache’* (Twohig et al. [20]). Others, however, recognized that their condition was fragile: *‘Another six months goes past, and I’m still not feeling any better… it’s a bit of a merry-go-round’* (Harris et al. [22]).

Participants described striving for a ‘new normal’ while fearing relapse and expressing disappointment at limited ongoing support: *‘I returned to my normal active life, but it came back… the hospital abandoned me’* (Harris et al. [22]). Across studies, living with PMR was characterized by continual adjustment-physically, emotionally and socially, as individuals attempted to balance acceptance with the pursuit of normality.

## Discussion

This qualitative narrative literature review exploring the patient experience of PMR has identified three main themes: (1) pathway to diagnosis, (2) managing uncertainty and (3) challenges to everyday life. These themes suggest potential opportunities to improve healthcare outcomes for patients with PMR.

This is the first paper of this kind to synthesize the different qualitative experiences of individuals with PMR described, in a manner that identifies themes and subthemes of patient experience. Despite differences in methodology, setting and sample size, the studies revealed consistent patterns in how patients make sense of and manage their condition, enabling a nuanced synthesis of how patients experience and manage PMR. They provide a broad and complementary perspective on the lived experience of PMR, reflecting diversity in age, gender and disease duration across both primary and secondary care settings in the UK and Australia.

The pathway to diagnosis theme could be addressed through motivating and educating patients to not normalize often very severe symptoms as part of the normal ageing process. More awareness about this condition is needed among both the general population and clinicians, including GPs, who are often the first point of call for symptoms, not just hospital doctors. Delay to diagnosis is also likely to be affected by current strains on healthcare services and delays in getting GP appointments. Although PMR accounts for up to a third of older people taking long-term low dose glucocorticoids, it is a relatively under-recognized condition and has many clinical masquerades [25]. This itself is confounded as the pathophysiology of PMR has not been fully elucidated, and further research investigating this could help the understanding and recognition of the disease.

Managing uncertainty around symptoms theme highlights the need for patient support in self-management, empowering individuals to navigate daily challenges, manage relapses and know when to seek help. Education is essential in informing those with a new diagnosis of PMR about both common and serious adverse effects of treatment as well as the symptoms of GCA and relapsing nature of the disease. Patients might be empowered to increase or decrease their daily glucocorticoid dose, in collaboration with healthcare professionals, but this could in turn add more psychological stress and patient responsibility. Additionally, clinicians may have concerns about clinical risks of self-adjusted dosing without a robust biomarker of disease activity.

Challenges to everyday life could be supported by the multidisciplinary team including occupational health and physiotherapy support, and by strengthening the emotional and social support available for those with PMR, with recognition that a better understanding of the patient’s views on their condition will improve the management of PMR symptoms [24]. This could be through individual based self-management techniques and programmes, and on a population level with support groups and organizations. Recognition of the challenges that PMR poses could be facilitated through different outcome measures for use in future studies in PMR including those that are more patient centric [26].

‘Relapse’ and ‘remission’ are concepts commonly used by medical professionals to describe worsening of disease activity (‘relapse’) and well-controlled disease (‘remission’). These concepts are often not viewed in the same way by patients with PMR, who experience their symptoms as part of their daily lives rather than as distinct stages and therefore may not recognize the overall trajectory of the disease. While clinicians strive to attain effective disease control at the lowest possible dose of medication, individual patients might prioritize either overall quality of life or glucocorticoid cessation [27]. Descriptions of the positive effects of glucocorticoids in theme 2 highlight that although there is an initial relief at diagnosis in glucocorticoid-responsive disease, this can soon be replaced by a sense of anxiety and poorly controlled disease if the glucocorticoid dose is insufficient or reduced too quickly. This brings patients back to how they felt before their diagnosis and exacerbates the psychosocial burdens outlined in theme 3. This demonstrates the interplay between these themes and the consequences for the patients.

Limitations of this qualitative narrative review include that studies were only carried out in the UK and Australia and therefore do not represent non-Western populations and selection bias cannot be ruled out. Furthermore, narrative reviews inherently rely on authors judgement in selecting and interpreting the literature and therefore do not offer the same level of transparency or reproducibility as systematic reviews. Other limitations relate to the confounding effects of the related condition, GCA. GCA was included in the search criteria to capture all relevant PMR cases, but studies focusing solely on GCA without mention of PMR were excluded. Due to the overlap between GCA and PMR, the search criteria may not be able identify all relevant published experiences of the disease due to classification bias. However, by inclusion of GCA, we facilitate the identification of additional cases of PMR, and this was therefore prioritized. One of the five studies (Harris et al. [22]) had limited data reporting, and this challenge was addressed by securing ethics and permissions to access full data transcripts from the authors.

The result of this qualitative narrative review demonstrates that studies of lived experience of individuals living with PMR are a relatively neglected area of study. However, we feel we have been able to reveal rich patient insights and develop meaningful themes that authentically represent the experiences of individuals living with PMR and explore the available studies carried out over a variety of different clinical settings and geographical locations. This review contributes to the understanding of individuals’ health-seeking behaviour, the nature of PMR and the impact of treatment on patients’ everyday lives. Furthermore, the findings suggest that relapse and remission criteria should incorporate the lived experience of PMR, recognizing the interplay of physical, functional and psychological dimensions that shape how patients perceive and manage disease activity. These unique patient insights should inform healthcare decision-making and guide the development of tools to measure disease activity, ultimately enhancing the quality of life for individuals living with PMR.

## Conclusion

This qualitative narrative literature review, although based on a limited number of studies, synthesized existing research on the lived experience of PMR that offer an understanding of how individuals experience the disease. The review highlights the need for greater public awareness of PMR to support earlier help-seeking. Rapid access to clinical support, particularly around the management of glucocorticoid therapy, is essential. Close monitoring during glucocorticoid tapering is also critical to prevent relapse and ensure responsive, adaptive care. Equally, this review underscores the importance of validating patient experiences and addressing the psycho-social burden PMR places on daily life. Ultimately, these insights call for a more integrative, person-centred approach to PMR management—one that extends beyond initial diagnosis and provides continuous, holistic support throughout the disease course.

## Supplementary Material

rkag006_Supplementary_Data

## Data Availability

All data in this review were obtained from published literature or by contacting study teams.
