# Supplementary material for: Patient experiences of PMR: a qualitative narrative literature review
Source: Rheumatol Adv Pract. 2026 Jan 16;10(1):rkag006. doi: 10.1093/rap/rkag006 (PMC12883660; doi:10.1093/rap/rkag006)
Supplement: rkag006_Supplementary_Data [file rkag006_supplementary_data.docx]

**Supplementary Table S1 – Critical Appraisal Skills Programme (CASP) Criterion for Quality Appraisal**

| **1^st^ Author (year of study publication)** | **CASP criterion** | | | | | | | | | | |
| --- | --- | --- | --- | --- | --- | --- | --- | --- | --- | --- | --- |
|  | **1** | **2** | **3** | **4** | **5** | **6** | **7** | **8** | **9** | **10** | **Total Score** |
| Tshimologo et al (2017) | 2 | 2 | 2 | 2 | 2 | 2 | 2 | 2 | 2 | 2 | 20 |
| Twohig et al (2015) | 2 | 2 | 2 | 2 | 2 | 2 | 2 | 2 | 2 | 2 | 20 |
| Hoon et al (2019) | 2 | 2 | 2 | 2 | 2 | 2 | 2 | 2 | 2 | 2 | 20 |
| Mackie et al (2015) | 2 | 2 | 2 | 2 | 2 | 2 | 2 | 2 | 2 | 2 | 20 |

Question index:

1. Was there a clear statement of the aims of the research?

2. Is a qualitative methodology appropriate?

3. Was the research design appropriate to address the aims of the research?

4. Was the recruitment strategy appropriate to the aims of the research?

5. Was the data collected in a way that addressed the research issue?

6. Has the relationship between researcher and participants been adequately considered?

7. Have ethical issues been taken into consideration?

8. Was the data analysis sufficiently rigorous?

9. Is there a clear statement of findings?

10. How valuable is the research?

Quality Scoring:

- **0–10** → low quality
- **11–15** → medium quality
- **16–20** → high quality
